# Laser Doppler Flowmetry Combined with Spectroscopy to Determine Peripheral Tissue Perfusion and Oxygen Saturation: A Pilot Study in Healthy Volunteers and Patients with Peripheral Arterial Disease

**DOI:** 10.3390/jpm12060853

**Published:** 2022-05-24

**Authors:** Kirsten F. Ma, Simone F. Kleiss, Richte C. L. Schuurmann, Thomas S. Nijboer, Mostafa El Moumni, Reinoud P. H. Bokkers, Jean-Paul P. M. de Vries

**Affiliations:** 1 Division of Vascular Surgery, Department of Surgery, University of Groningen, University Medical Center Groningen, 9713 GZ Groningen, The Netherlands; s.f.kleiss@umcg.nl (S.F.K.); r.c.l.schuurmann@umcg.nl (R.C.L.S.); t.s.nijboer@umcg.nl (T.S.N.); j.p.p.m.de.vries@umcg.nl (J.-P.P.M.d.V.); 2 Division of Trauma Surgery, Department of Surgery, University of Groningen, University Medical Center Groningen, 9713 GZ Groningen, The Netherlands; m.el.moumni@umcg.nl; 3 Department of Radiology, Medical Imaging Center, University of Groningen, University Medical Center Groningen, 9713 GZ Groningen, The Netherlands; r.p.h.bokkers@umcg.nl

**Keywords:** diffuse reflectance spectroscopy, endovascular procedures, laser Doppler flowmetry, peripheral arterial disease, tissue perfusion

## Abstract

Background: In this study, we assessed the ability of the EPOS system (Perimed AB, Järfälla, Stockholm, Sweden) to detect differences in tissue perfusion between healthy volunteers and patients with peripheral arterial disease (PAD) with different severity of disease. Methods: This single-center prospective pilot study included 10 healthy volunteers and 20 patients with PAD scheduled for endovascular therapy (EVT). EPOS measurements were performed at rest at 32 °C and 44 °C, followed by transcutaneous oxygen pressure (TcPo_2_) measurements. The measurements were performed on the dorsal and medial side of the foot, as well as the lateral side of the calf. EPOS parameters included hemoglobin oxygen saturation (HbSo_2_) and speed-resolved red blood cell (RBC) perfusion. Results: HbSo_2_ at 44 °C was significantly different between the three groups for all measurement locations. The overall speed-resolved RBC perfusion at 44 °C was statistically significant between the groups on the dorsal and medial side of the foot but not on the calf. TcPo_2_ values were not significantly different between the three groups. Conclusions: This study demonstrates that the EPOS system can depict differences in tissue perfusion between healthy volunteers, patients with Fontaine class IIb PAD, and those with Fontaine class III or IV PAD but only after heating to 44 °C.

## 1. Introduction

Noninvasive measurement of tissue perfusion may assist in the early diagnosis and assessment of impaired tissue perfusion in patients with peripheral arterial disease (PAD) [1,2]. One of these techniques is laser Doppler flowmetry (LDF), which can be used in combination with a provocation test to evaluate vascular reactivity in PAD patients [3,4,5]. Previous studies investigating LDF used provocation protocols, such as local heating and cuff occlusion with hyperemic responses, to examine microvascular reactivity in the lower extremity [6,7,8]. These studies focused on tissue perfusion measurements with laser Doppler; however, information on hemoglobin oxygen saturation (HbSo_2_) is lacking, which can be measured with techniques that use spectroscopy.

A new optical method that uses LDF in combination with diffuse reflectance spectroscopy (DRS) for microcirculatory assessment of tissue is the PeriFlux 6000 enhanced perfusion and oxygen saturation (EPOS) system (Perimed AB, Järfälla, Stockholm, Sweden) [9,10,11]. DRS uses white light in the visible to near-infrared range and is able to calculate the HbSo_2_ and concentration of red blood cells (RBCs). By combining both systems in one probe, this device enables measurement of HbSo_2_ simultaneously with RBC tissue fraction and speed-resolved RBC perfusion in different speed ranges. The ability to determine the status of tissue perfusion of the lower extremity within PAD patients has not been investigated with the EPOS system to date.

In this pilot study, we investigated the feasibility of measurements based on the EPOS system and assessed whether the EPOS system can detect differences in tissue perfusion between healthy volunteers and patients with PAD of differing disease severity. Measurements were performed with and without heat provocation. As a secondary aim, we investigated the correlation between one of the parameters from the EPOS system with conventional transcutaneous oxygen pressure (TcPo_2_).

## 2. Materials and Methods

This single-center prospective pilot study included 10 healthy volunteers and 20 patients with PAD scheduled for endovascular therapy (EVT), comprising 10 patients classified as Fontaine class IIb and 10 patients classified as Fontaine class III or IV. Participants were included between September 2020 and April 2021 at the University Medical Center Groningen (UMCG).

The Institutional Review Board approved the study (IRB #2019/517), and all participants provided individual written informed consent. Study procedures were performed according to the Medical Research Involving Human Subjects Act and the Declaration of Helsinki. The study was registered in the Netherlands Trial Register (#NL8023).

### 2.1. The EPOS System

The model-based analysis within the EPOS system is a combination of the LDF and DRS spectra [12,13]. The frequency content in the LDF spectra originates from Doppler shifts of laser light (785 nm) when scattered by moving RBCs. DRS measures white light in the visible to near-infrared range of 475 to 750 nm. Because hemoglobin has clear oxygen-dependent absorption spectra, it is possible to calculate the HbSo_2_ and concentration of RBCs. The EPOS system is also able to measure speed-resolved RBC perfusion within three different speed ranges of interest (0–1 mm/s, 1–10 mm/s, and >10 mm/s) [11,12]. The algorithms in the EPOS system are based on machine learning; the training is based on simulated DRS and LDF spectra from complex tissue-relevant models, resulting in fast and robust calculation of the output parameters [13].

The average sampling depth of the EPOS system is 0.5 to 0.7 mm, [14,15] which includes the epidermal and dermal layers, with capillaries, arterioles, and venules present in the latter. In the literature, higher-speed ranges have been associated with higher blood flow speeds of larger vessels and lower speeds with lower blood flow speeds of smaller vessels [15]. Tissue perfusion measurements with the EPOS system can be performed by heating the optical probe to different predefined temperatures. The system has been validated and measures the following perfusion parameters: HbSo_2_, RBC tissue fraction, and speed-resolved RBC perfusion within three different regions of speed [9,16].

### 2.2. Study Population

Healthy volunteers were recruited among visitors and employees of the UMCG. Inclusion criterion for healthy volunteers was age ≥ 50 years. Exclusion criteria were illiteracy or language barrier; symptoms or history of PAD; ankle-brachial index (ABI) < 0.90; peripheral edema; leg, ankle, or foot fractures within the past 12 months; dermatologic diseases influencing measurements; cellulitis, erysipelas, or other infections of the lower extremity; neurologic diseases; diabetes mellitus; and current or former cardiovascular disease. Baseline characteristics, including age, sex, body mass index (BMI), and smoking status, were recorded.

Patients with PAD were screened for eligibility when visiting the outpatient clinic at the vascular surgery department. Inclusion criterion was age ≥ 18 years. A total of 10 patients with Fontaine class IIb PAD and 10 patients with Fontaine class III or IV PAD were selected for inclusion. Exclusion criteria were illiteracy or language barrier; recent leg, ankle, or foot fractures; dermatologic diseases influencing measurements; and severe peripheral edema, cellulitis, erysipelas, or other infections of the treated leg or foot.

Baseline patient characteristics, including age, sex, BMI, smoking status, comorbidities, ABI, and Fontaine classification, were collected from medical records. A multidisciplinary team of vascular surgeons and interventional radiologists determined the Fontaine classification and the indication for EVT based on clinical evaluation, Doppler ultrasound imaging, and computed tomography angiography.

All treated lesions in the patients with PAD were scored according to the trans-Atlantic inter-society consensus for management of peripheral arterial disease (TASC-II) classification by the operating interventional radiologist [17]. For patients with Fontaine class III or IV PAD, the infrainguinal and inframalleolar global limb anatomic staging system (GLASS) score was also evaluated by the interventional radiologist based on digital subtraction angiography during EVT [18].

### 2.3. Measurement Protocol

A standardized measurement protocol was developed to perform perfusion measurements with the EPOS system at the lower extremity (Supplementary Materials Figure S1). The feasibility and clinical performance of the EPOS system were practically assessed within this measurement protocol [19]. Practical feasibility was assessed with attention to adequate probe placement, quality of received signal, and time to plateau phase in the EPOS signal. All participants underwent protocol measurements at the outpatient clinic or the surgical ward.

**Figure 1:**
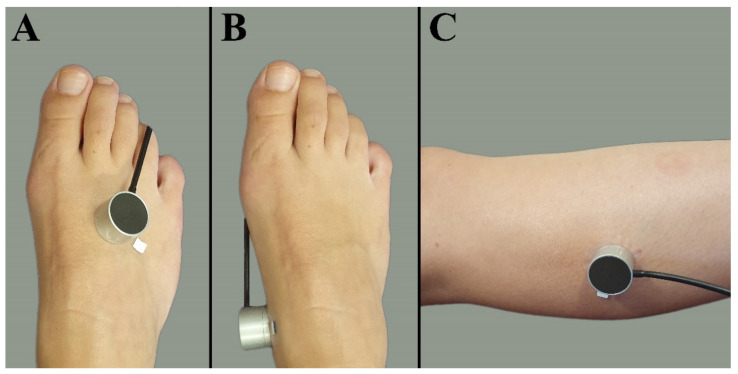
Measurement locations at the lower extremity. (**A**) Dorsal side of the foot. (**B**) Medial side of the foot. (**C**) Lateral side of the calf muscle 5 cm distally from the fibular head.

Patients with PAD underwent the measurements 1 day before EVT. Participants were placed on an examination bed in semi-Fowler’s position with knees extended. Measurements were conducted in a climate-controlled room with a mean ± SD ambient temperature of 21.9 ± 1.3 °C. All participants rested at least 5 min before recordings started at the first measurement location. Tissue perfusion measurements were performed at three measurement locations on the lower extremity: the dorsal side of the foot, the medial side of the foot, and the lateral side of the calf, 5 cm distally from the fibular head. A random leg was chosen in the healthy volunteers, whereas the treated leg was measured in patients. If patients had ulcers near one of the measurement locations, the probe was placed on surrounding skin without edema. The probe was attached to the skin with a double adhesive ring (PF 105-1, Perimed AB), avoiding any visible blood vessel, tendon, or bone. An example of the EPOS probe attached on the three different measurement locations is shown in Figure 1.

The EPOS measurements started on the dorsal side of the foot with a probe of 32 °C for at least 3 min. This was repeated for the medial side of the foot and the calf. The exact placement of the EPOS probe was marked with a pen on the double adhesive ring, which was not removed from the skin during measurements. After the EPOS probe was removed from the adhesive ring of the calf, measurements were performed with heat provocation by increasing the temperature to 44 °C at the exact same locations. These measurements lasted 15 min per location. The entire EPOS measurement protocol lasted 65 min.

After the EPOS measurements were completed, TcPo_2_ measurements were performed at the same measurement locations. The TcPo_2_ measurements were performed with a PeriFlux 6000 TcPo_2_ system (Perimed AB, Järfälla, Stockholm, Sweden). The TcPo_2_ sensor was attached and heated to 44 °C, and measurements were performed for 15 min. Blood pressure, heart rate, arterial saturation, and ABI were determined after the measurement protocol was completed. Researchers were trained by Perimed AB for use of the EPOS and TcPo_2_ systems.

### 2.4. Data Analysis of the EPOS Parameters

**Figure 2:**
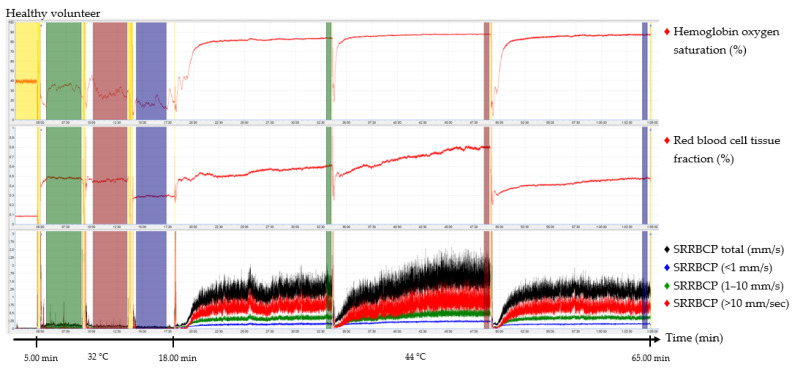
Continuous enhanced perfusion and oxygen saturation (EPOS) values of one healthy volunteer. Green times of interest (TOIs) are measurements on the dorsal side of the foot. Red TOIs are measurements on the medial side of the foot. Blue TOIs are measurements on the calf. Yellow regions are motion artifacts. All participants rested at least 5 min before recordings started. SRRBCP: speed-resolved red blood cell perfusion.

**Figure 3:**
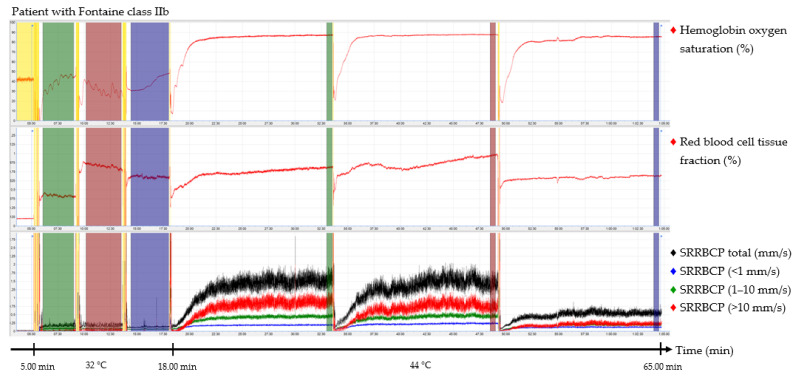
Continuous enhanced perfusion and oxygen saturation (EPOS) values of one patient with Fontaine class IIb. Green times of interest (TOIs) are measurements on the dorsal side of the foot. Red TOIs are measurements on the medial side of the foot. Blue TOIs are measurements on the calf. Yellow regions are motion artifacts. All participants rested at least 5 min before recordings started. SRRBCP: speed-resolved red blood cell perfusion.

**Figure 4:**
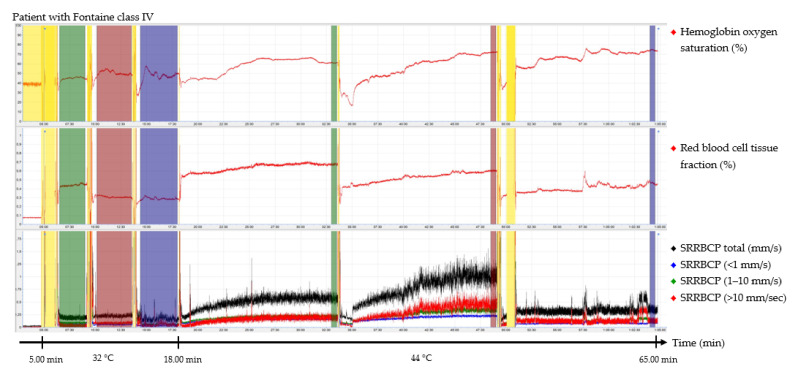
Continuous enhanced perfusion and oxygen saturation (EPOS) values of one patient with Fontaine class IV. Green times of interest (TOIs) are measurements on the dorsal side of the foot. Red TOIs are measurements on the medial side of the foot. Blue TOIs are measurements on the calf. Yellow regions are motion artifacts. All participants rested at least 5 min before recordings started. SRRBCP: speed-resolved red blood cell perfusion.

The output parameters of the EPOS system were HbSo_2_ (%), RBC tissue fraction (g RBC/100 g tissue or %), and speed-resolved RBC perfusion in percentage of RBCs times their speed in mm/s (% RBC × mm/s). The mean hemoglobin concentration per RBC was assumed to be 345 g/L [15]. Examples of the EPOS measurements in a healthy volunteer, a patient with Fontaine class IIb PAD, and a patient with Fontaine class IV PAD are shown in Figure 2, Figure 3 and Figure 4, respectively.

Times of interest (TOIs) were drawn over the EPOS signals to calculate mean values for specific durations. The TOIs of the measurements at 32 °C were placed over the complete time intervals due to fluctuation of the signal at a temperature of 32 °C. TOIs of 30 s of the measurements at 44 °C were placed at the end of the 15 min measurement period to ensure a measurement with sufficient heating of the skin. Placement of the probe within the holder during the rest period of the patient and motion artifacts due to displacement of the EPOS probe to a different measurement location were excluded from the TOIs. An example of this is shown in the measurement of Figure 4 in the green TOI on the dorsal side of the foot at 32 °C, where the TOI was shortened. The time difference between the three groups to reach a perfusion plateau was measured by calculating the time to reach 90% of the HbSo_2_ value of the measured TOI.

### 2.5. Statistical Analysis

Data were recorded using case report forms in an electronic database, REDCap (Vanderbilt University, Nashville, TN, USA). Statistical analysis was performed using SPSS 23 software (IBM Corp., Armonk, NY, USA). Descriptive statistics are presented as median with interquartile range (IQR; 25th and 75th percentile) according to the data distribution. Differences between the three groups (healthy volunteers, patients with Fontaine class IIb PAD, and patients with Fontaine class III or IV PAD) were tested using a Kruskal–Wallis test. Correlation between HbSo_2_ and TcPo_2_ was determined with a Spearman correlation coefficient. A correlation coefficient of 0.0 to 0.1 was considered negligible, 0.1 to 0.39 was considered weak, 0.40 to 0.69 was considered moderate, 0.7 to 0.89 was considered strong correlation, and 0.9 to 1.0 was considered a very strong correlation [20]. For analysis, *p* values ≤ 0.05 were considered statistically significant.

## 3. Results

### 3.1. Characteristics of Participants

Participant characteristics are summarized in Table 1. Four patients with Fontaine class III PAD and six patients with Fontaine class IV PAD were included in the third group. The median age was significantly lower in healthy volunteers (55.0 (IQR 53.8–60.0) years) compared with the Fontaine class IIb group (66.5 (IQR, 59.3–74.0) years) and the Fontaine class III and IV group (70.5 (IQR, 67.3–76.3) years, *p* = 0.001). Both patient groups had a high prevalence of typical vascular comorbidities, such as hypertension and coronary artery disease. Blood pressure, heart rate, arterial saturation, ABI, TcPo_2_, TASC-II classification, and GLASS classification are presented in Table 2. ABIs of five patients were missing, two due to noncompressible vessels, two due to ulcers, and due to logistical reasons in a patient with Fontaine class IIb PAD. ABI was 1.22 (IQR, 1.07–1.37) for healthy volunteers, 0.78 (IQR, 0.67–0.84) for the Fontaine class IIb group, and 0.52 (IQR, 0.49–0.69) for the Fontaine class III or IV group, and these differences were statistically significant (*p* < 0.001). TcPo_2_ values were not significantly different between the three groups.

### 3.2. Feasibility and Clinical Performance

No problems occurred with probe placement of the EPOS and TcPo_2_ systems on the dorsal side of the foot and on the calf in any of the participants. Due to the curvature of the medial side of the foot, release of the TcPo_2_ electrode occurred in four patients, resulting in four (13.3%) missing values. One TcPo_2_ value on the calf was missing because of technical failure.

Signals of EPOS parameters were of good quality without motion artifacts or fluctuations on the dorsal side of the foot and calf in all participants. EPOS parameters showed highly fluctuating signals on the medial side of the foot in one healthy volunteer and one patient with Fontaine class IIb PAD, accounting for 6.7% of the measurements on the medial side of the foot. These fluctuations could be an effect of vasomotion, the spontaneous oscillation in vascular tone in the microcirculation, which was observed in the EPOS parameters [21]. None of these measurements were excluded from the data analysis.

TOIs for 32 °C EPOS measurements had an average duration of 3:20 min after removal of motion artefacts caused by moving the probe or temporal movement of the leg from all three measurement locations. A duration of 15 min for measurements at 44 °C was sufficient to reach a plateau phase in all healthy volunteers and patients with Fontaine class IIb PAD; however, in six measurements (20%) of patients with Fontaine class III or IV PAD, the signal was still increasing after 15 min. Motion artefacts were also removed from the 44 °C EPOS measurements.

### 3.3. Perfusion Quantification

**Figure 5:**
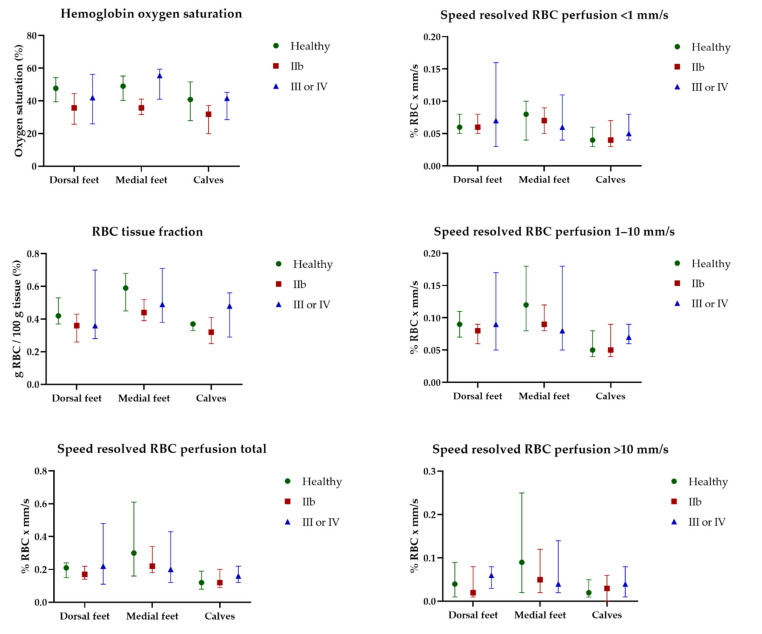
Continuous enhanced perfusion and oxygen saturation (EPOS) variables at 32 °C between healthy volunteers, patients with Fontaine class IIb, and patients with Fontaine class III or IV measured on the dorsal side of the foot, medial side of the foot, and the lateral side of the calf. Data are presented as median (interquartile range). Healthy: healthy volunteer, RBC: red blood cell, IIb: patients with Fontaine class IIb, IV: patients with Fontaine class III or IV.

**Figure 6:**
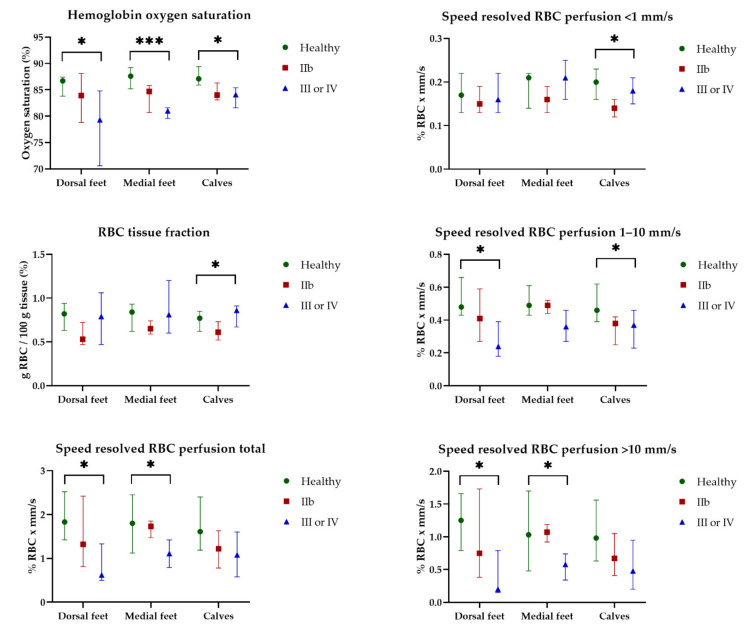
Continuous enhanced perfusion and oxygen saturation (EPOS) variables at 44 °C between healthy volunteers (Healthy), patients with Fontaine class IIb (IIb), and patients with Fontaine class III or IV (III or IV) measured on the dorsal side of the foot, medial side of the foot, and the lateral side of the calf. Data are presented as median (interquartile range). Statistical significance: * *p* < 0.05, *** *p* < 0.001.

HbSo_2_, RBC tissue fraction, and speed-resolved RBC perfusion at 32 °C for all participants are presented in Figure 5. EPOS parameters at 32 °C did not differ significantly for any of the three measurement locations between the three groups. There was an increase in all EPOS parameters after local heating to 44 °C compared with 32 °C at all measurement locations for all participants. EPOS variables at 44 °C measured on the dorsal side of the foot, medial side of the foot, and the lateral side of the calf in all three groups are shown in Figure 6. Median HbSo_2_ on the dorsal side of the foot was 86.7 (IQR 83.8–87.4) for healthy volunteers, 83.9 (IQR 78.8–88.1) for patients with Fontaine class IIB, and 79.3 (IQR 70.6–84.8) for patients with Fontaine class III or IV (*p* = 0.019). Median HbSo_2_ was significantly different at 44 °C between the three groups for the medial side of the foot 87.6 (IQR 85.2–89.2) for healthy volunteers, 84.7 (IQR 80.7–85.8) for patients with Fontaine IIB, and 81.0 (IQR 79.6–81.6) for patients with Fontaine class III or IV, (*p* < 0.001). Median HbSo_2_ on the calf was 87.1 (IQR 85.9–89.4) for healthy volunteers, 84.0 (IQR 83.1–86.3) for patients with Fontaine class IIB, and 84.1 (IQR 81.6–85.4) for patients with Fontaine class III or IV (*p* = 0.022).

Speed-resolved RBC perfusion total also showed a difference between the three groups and was statistically different on the dorsal (*p* = 0.016) and medial (*p* = 0.043) sides of the foot but not on the calf (*p* = 0.057). Among the speed ranges, the lowest speed region (<1 mm/s) did not show a difference between the groups; however, in the higher speed ranges (1–10 mm/s and >10 mm/s), a statistically significant decrease was observed in both speed ranges for the dorsal side of the foot (*p* = 0.023 and *p* = 0.015, respectively).

HbSo_2_ showed a moderate correlation with TcPo_2_ measurements on the dorsal side (R = 0.470, *p* = 0.009) and medial side (R = 0.468, *p* = 0.016) of the foot and a weak correlation on the calf (R = 0.240, *p* = 0.209).

The median time to reach 90% of the HbSo_2_ value of the measured TOI was 92.0 s (IQR, 56.8–112.5 s) for healthy volunteers on the dorsal side of the foot, 46.5 s (IQR, 27.3–60.3 s) on the medial side of the foot, and 68.5 s (IQR, 50.8–84.5 s) on the calf. For patients with Fontaine class IIb PAD, the median time to reach 90% was 102.0 s (IQR, 59.5–169.5 s), 69.5 s (IQR, 36.0–92.5 s), and 110.0 s (IQR, 83.5–136.0 s), respectively. For patients with Fontaine class III or IV PAD, median time intervals were 262.0 s (IQR, 112.0–345.8 s), 281.0 s (IQR, 35.8–421.5 s), and 101.0 s (IQR, 73.0–146.8 s), respectively. Differences of time intervals between the groups were statistically significant for the dorsal side of the foot (*p* = 0.024) and the calf (*p* = 0.007) but not for the medial side of the foot (*p* = 0.170).

## 4. Discussion

This study demonstrates that the EPOS system is able to detect differences in tissue perfusion between healthy volunteers, patients with Fontaine class IIb PAD, and patients with Fontaine class III or IV PAD. Differences in tissue perfusion between the groups were only detected after heating to 44 °C, emphasizing the need for a heat provocation test in patients with PAD when tissue perfusion is measured at rest. Differences between the groups were not detected with TcPo_2_.

Previous studies investigating the EPOS system were limited to the validation of the system in the skin properties of 1734 healthy volunteers [9,10]. EPOS is a potentially interesting technique to monitor tissue perfusion because it is noninvasive, can be implemented throughout the entire periprocedural process in patients with PAD, and is able to detect differences in microcirculation status, which is essential for the diagnosis of PAD, especially in patients with ulcers or gangrene. EPOS measurements were feasible in all patients included in the study, in contrast with ABI, which could not be performed in patients with ulcers or non-compressible vessels. A similar technique used in PAD is microlightguide spectrophotometry, also called oxygen to see (O2C). The difference between the systems is the algorithms used, which is the reason tissue fraction and perfusion in EPOS are measured in absolute units instead of relative units, as in O2C [22,23].

An additional feature of the EPOS system may be the measurement of perfusion within different speed ranges, which is not possible with the O2C method. Jørgensen et al. examined O2C in 59 participants with and without PAD and showed no significant differences at baseline comparing HbSo_2_ and flow parameters. When the limb was elevated, however, patients without PAD had a significantly higher HbSo_2_ [22]. These findings are comparable with those findings of the present study.

In this study, an increase in all EPOS parameters after local heating was determined. It seems that the increase in temperature and therefore the increase in tissue perfusion (HbSo_2_) is higher than the increase in tissue consumption. Local heating causes maximum local hyperemia, which enables measurement of the tissue arterial capacity. Patients with PAD with low arterial capacity have a lower maximum compared with healthy volunteers, which is demonstrated the higher speed ranges of the speed-resolved RBC perfusion parameters of this study. Limb elevation was not applied in this study, its relationship with heat provocation is unclear.

Jonasson et al. presented the first results in patients with diabetes mellitus and healthy individuals with an early prototype of the EPOS system [24]. They showed significant differences between healthy volunteers and patients with diabetes with and without microalbuminuria in low-speed (<1 mm/s) resolved RBC perfusion at 32 °C and 44 °C.

Despite of the heterogeneity of the groups included in this study, the EPOS system was able to differentiate between the three groups in only 30 participants. The largest differences were determined in the higher speed ranges of speed-resolved RBC perfusion (1–10 mm/s, >10 mm/s) but not in the low-speed range (<1 mm/s). The discrepancy between our results and previous findings could be the result of differences between patients with PAD and those with diabetes. We hypothesize that stenosis or occlusions in larger arteries affect the highest speeds measured with the EPOS system at most, although EPOS does not directly measure larger arteries. The complexity of the dependence of the speed of blood flow on external and internal factors should be taken into account (e.g., viscosity, vessel wall resistance, size of the lumen, and timing of the cardiac action). In our study, we focused on the first 0.5–0.7 mm of the skin, which excludes the largest vessels.

We determined EPOS parameters with TOIs and calculated the time to reach 90% of the HbSo_2_ value of the measured TOI at three different locations of the lower extremity. TOIs of 30 s at the end of 15 min were chosen to standardize the protocol between the three groups. The median time to reach 90% of the maximum HbSo_2_ value showed a large IQR on the dorsal and medial sides of the foot, probably due to the small group of patients included in this study, and should be determined in larger future studies. The 15 min measurements at 44 °C were sufficient to reach a plateau phase in all healthy volunteers and patients with Fontaine class IIb PAD; however, in 20% of the measurements in patients with Fontaine class III or IV PAD, the signal was still increasing after 15 min. Measurement time of healthy volunteers and patients with Fontaine class IIb PAD should be shortened in future studies and extended in patients with chronic limb-threatening ischemia (CLTI). Explaining the extended time to reach 90% in this patient group is difficult, given that the endothelial or neurosympathetic response to warming in the vessels was not assessed. This parameter seems important for measuring tissue perfusion due to large differences between the groups. Reekers et al. investigated the capillary resistance index to measure the response of the sympathetic nervous system in patients with diabetes, which may be a useful tool in future studies [25].

A weak to moderate correlation was observed between TcPo_2_ and HbSo_2_, which is contrary to previous findings, where no correlation was found in the calves of healthy volunteers at rest [26]. It should be noted that TcPo_2_ and HbSo_2_ are measurements of two different physiological quantities; the former reflects the free oxygen in the tissue that has diffused from the blood through microcirculation, whereas the latter reflects the oxygen bound to hemoglobin in through microcirculation.

One of the limitations of this study is the small group of 20 patients with PAD, including patients with and without diabetes in both Fontaine groups. Healthy volunteers had a younger age and fewer comorbidities compared to patients with PAD, which is a limitation of the studied population. Patients with diabetes with a disturbed microvascular regulation are assumed to have an altered hyperemic response to local heating compared with patients without diabetes. In addition, the patient group with Fontaine class III or IV PAD was a heterogeneous group with differing severity of PAD. Patients were selected based on a scheduled EVT with corresponding Fontaine class. The Fontaine classification, however, does not include an objective measure, such as ankle pressure, toe pressure, or wound classification. Toe pressures or ankle pressures were not available for all patients in this study within medical records. For these reasons, not all patients in this group can be verified as CLTI. This may be one of the reasons that no statistical differences were found in TcPo_2_ between the three groups [27]. However, the heterogeneity in this group is a relevant reflection of daily clinical practice. The influence of the small sample size could also be a reason for the lack of significant statistical differences in TcPo_2_ and EPOS parameters at 32 °C, which may present differences a study with a larger sample size.

Finally, results on the medial side of the foot should be interpreted with caution because the measurements with fluctuations due to vasomotion were not excluded, and probe placement was more difficult. The dorsal sides of the foot and the calf are recommended as the most practical locations for perfusion measurements with the EPOS and TcPo_2_ systems.

Future studies should investigate the validity and reliability of tissue perfusion parameters with the EPOS with heat provocation in a more homogeneous and larger group of patients with CLTI throughout the entire periprocedural process. Standardized assessment with the Society for Vascular Surgery Wound, Ischemia, and foot Infection (WIFI) and Rutherford classifications is essential to better define CLTI. Future studies should also investigate whether the EPOS system is able to differentiate between patients with and without diabetic angiopathy and investigate whether tissue perfusion parameters are predictive of wound healing and amputation-free survival.

## 5. Conclusions

This study demonstrates that LDF combined with DRS in the EPOS system is able to differentiate between tissue perfusion at rest of healthy volunteers, patients with Fontaine class IIb PAD, and patients with Fontaine class III or IV PAD after heating to 44 °C. Differences were not detected without heat provocation. HbSo_2_ showed a weak to moderate correlation with TcPo_2_ measurements.

## Figures and Tables

**Table 1:** Characteristics of included healthy volunteers, patients with Fontaine class IIb, and patients with Fontaine class III or IV.

	Healthy Volunteers	Patients with Fontaine Class IIb	Patients with Fontaine Class III or IV
	*n* = 10	*n* = 10	*n* = 10
Age (y)	55.0 (53.8–60.0)	66.5 (59.3–74.0)	70.5 (67.3–76.3)
Sex			
− Female	4 (40%)	6 (60%)	5 (50%)
− Male	6 (60%)	4 (40%)	5 (50%)
Body mass index (kg/m^2^)	24.0 (22.4–27.2)	26.7 (24.5–30.8)	26.6 (21.5–29.6)
Smoking			
− Smoker	0 (0%)	4 (40%)	5 (50%)
− Ex-smoker	2 (20%)	5 (50%)	5 (50%)
− Non-smoker	8 (80%)	1 (10%)	0 (0%)
Diabetes mellitus		1 (10%)	5 (50%)
− Type 1		0 (0%)	2 (20%)
− Type 2		1 (10%)	3 (30%)
Hypertension	1 (10%)	8 (80%)	5 (50%)
Coronary artery disease		7 (70%)	2 (20%)
Prior cerebral events		4 (40%)	1 (10%)
COPD		1 (10%)	2 (20%)
Renal dysfunction (eGFR < 60 mL/min/1.73 m^2^)		1 (10%)	4 (40%)
Hemodialysis			2 (20%)

Data are presented as median (IQR) or number (%). COPD = chronic obstructive pulmonary disease, eGFR = estimated glomerular filtration rate.

**Table 2:** Blood pressure, heart rate, saturation, ABI, TcPO2, TASC-II classification, and GLASS classification of healthy volunteers, patients with Fontaine class IIb, and patients with Fontaine class III or IV.

	Healthy Volunteers	Patients with Fontaine Class IIb	Patients with Fontaine Class III or IV	*p*-Value
	*n* = 10	*n* = 10	*n* = 10	
Blood pressure				
− Systolic	115.5 (111.0–120.3)	147.0 (119.0–156.5)	144.0 (131.3–156.8)	**0.005**
− Diastolic	78.5 (73.8–81.0)	65.5 (61.3–79.0)	69.0 (53.5–77.0)	0.072
Heart rate	56.0 (54.8–60.5)	66.0 (60.5–81.8)	75.5 (69.8–86.5)	**0.002**
Arterial saturation	97.0 (96.8–98.0)	98.0 (97.8–99.3)	98.0 (96.8–100.0)	0.115
Ankle-brachial index	*n* = 10	*n* = 8	*n* = 7	
	1.22 (1.07–1.37)	0.78 (0.67–0.84)	0.52 (0.49–0.69)	**<0.001**
TcPo_2_	*n* = 10	*n* = 10	*n* = 10	
− Dorsal side feet	57.0 (53.8–58.8)	60.2 (46.0–65.2)	50.7 (24.2–56.6)	0.191
	*n* = 9	*n* = 8	*n* = 9	
− Medial side feet	66.8 (63.7–72.4)	66.0 (59.4–76.9)	59.3 (44.2–66.6)	0.092
	*n* = 10	*n* = 10	*n* = 9	
− Calves	64.0 (56.4–72.2)	56.3 (50.8–69.1)	60.0 (47.9–71.0)	0.449
TASC-II classification				
− TASC-II A		4 (40%)	2 (20%)	
− TASC-II B		2 (20%)	3 (30%)	
− TASC-II C		1 (10%)	2 (20%)	
− TASC-II D		3 (30%)	3 (30%)	
GLASS classification				
Infrainguinal GLASS stage				
− N/A			1 (10%)	
− I			4 (40%)	
− II			2 (20%)	
− III			3 (30%)	
Inframalleolar				
− P0			2 (20%)	
− P1			3 (30%)	
− P2			5 (50%)	

Data are presented as median (IQR) or number (%). Differences between the three groups were tested with a Kruskal–Wallis test. EVT: endovascular therapy. *p*-values stated in bold indicate statistical significance of <0.05. The infrainguinal and inframalleolar global limb anatomic staging system (GLASS) score is based on digital subtraction angiography during EVT.

## Supplementary Materials

The following supporting information can be downloaded at: https://www.mdpi.com/article/10.3390/jpm12060853/s1, Figure S1: Flow chart of the study for all participants. EPOS: enhanced perfusion and oxygen saturation. TcPo_2_: Transcutaneous oxygen pressure measurement.
