# Supplementary material for: Laser Doppler Flowmetry Combined with Spectroscopy to Determine Peripheral Tissue Perfusion and Oxygen Saturation: A Pilot Study in Healthy Volunteers and Patients with Peripheral Arterial Disease
Source: J Pers Med. 2022 May 24;12(6):853. doi: 10.3390/jpm12060853 (PMC9224808; doi:10.3390/jpm12060853)
Supplement: Supplementary file 1 [file jpm-12-00853-s001.zip › jpm-1712115-supplementary.pdf]

Supplementary Figures

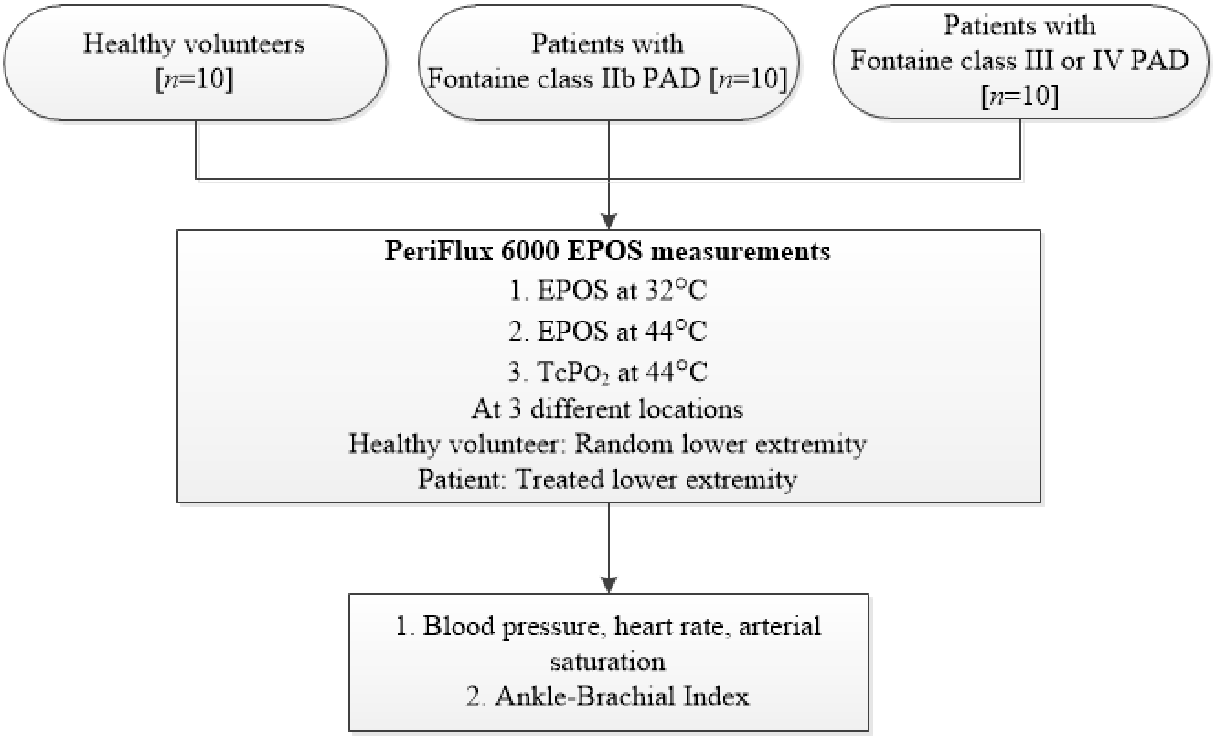

Supplementary Figure S1. Flowchart of the study for all participants. EPOS: Enhanced Perfusion and Oxygen Saturation. $TcPO_2$: Transcutaneous oxygen pressure measurement.
